# The Use of Sulphuric Acid in the Treatment of Roots for Immediate Filling

**Published:** 1895-03

**Authors:** Chas. Welch

**Affiliations:** Wilmington, O.


### The Use of Sulphuric Acid in the Treatment of Roots for Immediate Filling.

BY CHAS. WELCH, D.D.S., WILMINGTON, O.

Read before the Ohio State Dental Society, December, 1894.

Last year I was accused of bathing in sulphuric acid, which I deny, but every nerve canal that is treated by me, is bathed in it.

You want to know of my success? Under what conditions to expect a failure? How many I have treated? Manner of treatment? What teeth they were? Age of patients? What is accomplished in the way of cleansing, and in the stopping of hemorrhage? Is there any danger in allowing the acid to go through the apical foramen?

You remember Dr. Callahan's most able paper on this subject at our last meeting. He recommended the use of sulphuric acid in the opening of difficult root canals, but for straight and unobstructed ones used "Gates-Glidden drill." We now, after a year's successful experience, use the sulphuric acid in every case, thanking Dr. Callahan for the first thought or suggestion of this most useful aid to the profession.

Since December 11th, 1893, I have treated one hundred and three cases, and strange though it may seem, have been successful in every one of them. I filled every tooth immediately after using the acid fifty per cent. aqueous solution, excepting in cases of blind abscess with pus in the root canal. In those cases, after having cleansed the canal with acid, fill it with glycerine and iodoform made into a thick paste, leave it a week, then remove and fill permanently. That is the only condition I have found that you can not fill immediately. I have used this treatment upon every tooth in the mouth, without an exception, as a matter of experiment. Ages of patients ranging from fourteen to seventy-five years. Under every condition from a recently extracted live pulp to a chronic abscessed tooth.

In cases of hemorrhage in root canals sulphuric acid will always check it so you can fill immediately.

My manner of treatment is: First, apply the rubber dam. Open the pulp-chamber freely, take with pliers, a pledget of cotton soaked in sulphuric acid, and place it into the chamber. With a No 5 Donaldson Canal Cleanser pump the acid into the canal to the apex of the root. You may know when you have reached the apex by the patient complaining of pain. No trouble will arise by the acid going through the foramen. You know how dangerous it is to force a nerve broach through the foramen of any devitalized tooth, how soon you will have an alveolar abscess, but by using sulphuric acid, first on a pledget of cotton, it precedes the canal cleanser and kills the bacteria, preventing this trouble.

Second. With a drop tube flood the chamber with a saturated solution of bicarbonate of soda, which by the formation of carbonic acid gas throws out the debris, neutralizing the acid, at the same time cleanses and apparently whitens the tooth. Then dry thoroughly with a five per cent. solution of pyrozone followed with hot air.

Third. With a minim syringe flood the canal with carbolic acid and iodine, equal parts, followed with iodoform and gycerine which I force through the root. Then use chlora-percha with a gutta-percha point, which finishes the treatment, and the tooth is ready for a permanent filling.

### DISCUSSION.

Dr. J. R. Callahan: Dr. Welch goes a little beyond anything I have written on this subject when he gives details of after-treatment. That the treatment he announces has been successful in his hands is abundantly proven by the number of successful cases he reports. Although he does not mention the fact, I know that he has an accurate record of each and every one of the 103 cases that he reports. I thought at one time of having a large number of record cases reported at this meeting, but a friend and neighbor of mine talked me out of that idea, which perhaps was well enough, for Dr. Welch's record is sufficient, and it might be interesting to some of you to look it over.

I am often asked if I allow the acid to go through the foramen. My answer to this is that I *try* to get the acid through in every case. Of course I go very carefully. You should not ram the broach through the foramen as if you were drilling a hole in a rock; nor should you stab the instrument into the membranes beyond the root. With the *broach* you might do harm. The acid attacks vital tissues very mildly, but with devitalized tissue the attack is prompt and vigorous, so if the tissues about the apical space are in their normal condition, the presence of the small amount of acid that gets through the canal will create no disturbance further than a slight irritation, which will pass off in a very short time if you will prevent its becoming inoculated by the introduction of septic matter. If there be pus present, you will do well to flood the pus pocket with the acid solution.

A word of caution. When working in small canals, always take a new broach, and keep in mind that the acid makes the broach quite brittle.

After the root canals have been filled be sure to trim the margins of the cavity; that is cut away all the borders of the cavity that *might* have been touched by the acid, you need not cut deep, just enough to get a fresh surface for contact with the filling.

There is another thing I want to mention while on my feet, in cases of chronic abscess where it is necessary to amputate a portion of the root and remove dead bone. This operation leaves quite a chamber in the jaw that is often left to heal as best it may. This I believe to be wrong. This cavity should be packed full with iodoform gauze, and the gauze renewed at intervals of twenty-four to forty-eight hours, using a little less of the guaze at each visit. This is an old and established surgical practice.

Dr. O. N. Heise directed my attention to this after-treatment. Why it has been so generally overlooked is more than I can tell.

Dr. H. A. Smith: This discussion recalls the mineral acid theory of dental caries as taught us years ago by Professor George Watt.

He described three varieties of caries; one of which he claimed, was caused by sulphuric acid. By this acid the organic portion of the tooth is slowly carbonized, giving rise to what Professor Watt termed the black variety of dental caries. Accepting this theory as correct for the time, may we not produce by the use of sulphuric acid in root canal treatment artificial caries of Professor Watt's second variety?

Dr. Welch uses sulphuric acid in treating all root canals, whether septic or antiseptic. If the acid is used in foul root canals, may not a double purpose be effected? The canals would be widened by solution of the lime salts, and at the same time, the basis substance of the dentine, together with any micro-organisms present, would be burned and carbonized.

Perhaps the very general success which Dr. Welch claims has attended the use of sulphuric acid in putrefactive root canals, and in cases of abscessed teeth may be explained upon this hypothesis. It will be observed that in some of the cases reported by Dr. Welch, as cured by the sulphuric treatment, other well known antiseptics were used in connection with the acid. We are therefore left in doubt as to the real efficiency of this remedy, at least so far as these cases are concerned.

Dr. Harroun: Sulphuric acid is on record as the favorite treatment of Dr. W. H. Atkinson in all cases of necrosis, and it is no new treatment so far as cleansing the tissues is concerned. He used to tell us to use a quantity of vitriol, and not to be afraid of it. I have always made use of the aromatic sulphuric acid in such treatments.

Dr. Hoff: What has just been said about the use of elixer of vitriol is liable to lead us to confound this agent with dilute sulphuric acid, and cause confusion in results obtained. Elixer of vitriol is a twenty per cent. alcoholic solution of sulphuric acid and has no great power as a solvent of bone or soft tissues. This point was very satisfactorily settled by Dr. J. N. Farrar by a series of experiments recorded in the *Dental Cosmos* for 1878.

I see no special advantage in using the dilute sulphuric acid, in the treatment of decomposed pulps in the pulp chambers. It seems to me there are other agents which will just as effectively accomplish the removal of the dead soft tissues, and which have no objectionable qualities. Hot water is preferable, especially if a little soda and some good antiseptic be added. It is less objectionable, as it will not injure living tissues, the instruments, or patient's clothing, should an accident occur. Therefore, as a simple detergent, I would not make use of the sulphuric acid in such cases. I can, however, see no valid objection to one using the dilute sulphuric acid as a sterilizing agent and detergent if proper precautions are taken to protect the other tissues from its caustic properties.

Sulphuric acid is a cauterant, and is decomposed in the presence of water liberating oxygen, which destroys the molecular affinities of organized tissues. It is very active in destroying calcified tissues as well as soft tissues. I would use it in the treatment of pulpless teeth not primarily as a sterilizing agent, but as a detergent in a sense, that is to destroy the remains of the pulp organ and so much of the calcified tissues as was desirable to enlarge the pulp canal for free access.

My experience with it has been rather unfavorable. In one case I attempted to use it in the same way these gentlemen have described, but after destroying the pulp I couldn't remove it by ordinary detergents. A case in which I used it was one in which there was a large amount of bone deposited. The canal was almost entirely filled up, and I couldn't remove the pulp by the usual proceedings. It was very much irritated and inflamed, and I had much difficulty in trying to remove it. I injected cocaine and then drilled it out. I undertook the sulphuric acid treatment, and succeeded in enlarging the canal and removing the pulp entirely. I afterwards inserted a crown, and ever since the tooth has been giving trouble. There is no inflammation of the peridental membrane, but there is very much pain about the tooth. On testing it thoroughly, I am satisfied there is no inflammation about the tooth, but an irritated condition. I don't know whether it is due to treatment with sulphuric acid or not. Possibly it is. I am certain I did not encroach on the peridental membrane. I did encroach upon the cementum, and I can't account for it in that way. The absence of inflammation puzzles me a great deal about that tooth.

In regard to antiseptics following it, I think it is very good practice. I would use them for the reason that the sulphuric acid soon becomes diluted, although it may be an antiseptic and germicide in its active form.

In its undiluted form it is dissipated very quickly, because it is soluble in water and the fluids of the tissues. I think Dr. Welch's practice of using aristol is commendable, because it is not so quickly dissolved, remains a long time, giving the tissues a chance to recuperate under non-irritant antiseptic conditions.

